# Screening of Geriatric Depression in Makkah, Saudi Arabia: A Pilot Study

**DOI:** 10.7759/cureus.53313

**Published:** 2024-01-31

**Authors:** Hamsa AlQashqri, Nahla Hariri, Renad J Jadkarim, Alaa H Falemban, Enas Alfalogy

**Affiliations:** 1 Community and Family Medicine, Umm Al-Qura University, Makkah, SAU; 2 Community Medicine, Umm Al-Qura University, Makkah, SAU; 3 Epidemiology and Public Health, Umm Al-Qura University, Makkah, SAU; 4 Pharmacology and Toxicology, Umm Al-Qura University, Makkah, SAU; 5 Family Medicine, Suez Canal University, Ismailia, EGY; 6 Family Medicine, Umm Al-Qura University, Makkah, SAU

**Keywords:** risk factors, makkah, depression, elderly population, prevalence

## Abstract

Objective: There is currently limited evidence about the prevalence of depression among elderly people residing in Makkah, Saudi Arabia. This study aims to report the magnitude of depression among the older population in Makkah, Saudi Arabia, and the related risk factors.

Methods: An online cross-sectional pilot survey was carried out in Makkah City, Saudi Arabia. Data were collected using an online self-administered questionnaire.

Results: The study questionnaire was completed by 191 older people. The participants' ages varied from 60 to 88 years. 55.5% were women, 47.9% were married, and 21.5% were divorced/widowed. 46.6% had hypertension, 42.4% had diabetes, 17.3% had hypothyroidism, 7.9% had cardiovascular diseases (CVDs), and 6.3% reported psychiatric problems. 44.5% of the subjects had no depression, 23.5% had mild, 15.2% had moderate, and 16.8% had severe depression. The sample included 32% who had been classified as having major depression. Elderly participants with insomnia, cognitive diseases, and chronic diseases showed a high risk for experiencing severe depression (OR=2.74; 95% CI: 1.42-5.28),(OR=2.63; 95% CI: 1.29-5.40), and (OR=2.62; 95% CI: 1.11-6.14) respectively.

Conclusion: Depression was common among the elderly population in Makkah, particularly among those with a documented history of insomnia, cognitive diseases, and chronic diseases. Depression screening and treatment for old people in medical settings is recommended.

## Introduction

Late-life depression is the occurrence of major depressive disorder in adults 60 years of age or older [1,2]. In later life, depression is one of the most common and disabling psychiatric disorders. Depressive disorders are known to increase mortality and negatively affect the well-being and daily functioning of the elderly [3]. Its presence is also associated with increased medical morbidity and mortality, including higher than expected incidence of cardiovascular disease (CVD) events and stroke [4]. Most individuals with depression remain untreated or undertreated [5]. In several studies, it has been found that older adults with mental disorders are significantly less likely than younger adults to receive mental health care and treatment from mental health specialists. This may be due to the inability of older adults to verbalize their affective experiences and somatization, which masks their depression. Further, depression often goes unrecognized as a result of somatic comorbidity [3,6]. The USPSTF found good evidence that treating depressed adults and older adults identified through screening in primary care settings with antidepressants, psychotherapy, or both decreases clinical morbidity [4].

The rate of depression among the elderly population has increased in many studies locally and regionally. There is a wide range of prevalence rates of depression in Saudi Arabia, ranging from 12% to 49.9% [7-10]. Geriatric depression is prevalent in Arab nations as well, ranging from 20.2% in the United Arab Emirates [11] to 41.5% in Bahrain [12]. Several factors contribute to the development of depressive disorders, including female gender, single status, unemployment, somatic illnesses, pain, visual impairment, stroke, cognitive impairment, functional impairment, lack of close social contacts, frailty, poor oral intake, loneliness, lack of social support, adverse events in life, and perceived inadequacy of care [3,7-10].

To the best of our knowledge, there is minimal investigation on depression among elderly people of Makkah in Saudi Arabia in the general population. The objective of this study is to report the magnitude of depression among the older population in Makkah, Saudi Arabia, and the related risk factors.

## Materials and methods

Study setting and participants

An online cross-sectional pilot survey was conducted between the 1st of August and the 30th of November 2023 to report the magnitude and factors associated with depression among geriatric patients in Makkah City. The study's target population was all Saudi geriatric patients 60 years old and above, residing in Makkah City and accessing social media platforms, including WhatsApp or Telegram. Respondents were sent the online questionnaire accompanied by the objectives of the survey, the target population, and a request to participate voluntarily. The sampling strategy used in this study was a convenience sampling technique. Data was collected through an online Google Form containing the questionnaire in Arabic language and distributed to the participants.

Questionnaire

The questionnaire contained the following three sections: consent form; socio-demographic data, including age, gender, social status, education level, living with family, income, and employment; and depression questionnaire. The assessment of depression was conducted using the Patient Health Questionnaire (PHQ-9) [13]. Participants were queried on the frequency with which they experienced each symptom of depression in the last 14 days. The available responses included the phrase "not at all," "several days" (which refers to a number of days), "more than half the days" (which means a majority of the days), and "nearly every day" (which indicates almost all of the days). The scores were assigned as 0, 1, 2, and 3, respectively. The PHQ-9 scores have a range of 0 to 27. Depression severity levels were defined per the PHQ-9 scores as follows: mild if 5-9 points, moderate if 10-15 points, moderately severe if 15-19, and severe if more than or equal to 20 points. Major depression was defined as having a PHQ-9 score of more than or equal to 10 points. Psychometric properties of the Arabic version of PHQ-9 are valid and reliable in the Saudi population [14].

Sample size calculation

This study's minimum recommended sample size was 163 according to the software Sample Size Calculator (Raosoft, Inc., Seattle, WA, USA). With a population of elderly in Makkah city of 209,702 and estimating the prevalence of depression using the same instrument to be 12% [7], 163 participants were required to obtain a 95% confidence interval (i.e., that the obtained rate was within ±5%).

Ethical approval

The Institutional Research Board (IRB) at Umm Al-Qura University (HAPO-02-K-012-2023-05-1637) approved this study. Survey responses were collected anonymously.

Data analysis

The data were collected, reviewed, and then fed to STATA software. All statistical methods used were two-tailed with an alpha level of 0.05, considering the significance of P less than or equal to 0.05. Regarding PHQ-9, the overall score was obtained by summing up discrete scores for different items and then categorizing them into different severity levels about the given tool cut-off points.

Descriptive analysis was performed by prescribing frequency distribution and percentage for study variables, including participants' personal data, employment, medical data, and psychological or other cognitive disorders. Also, participants' responses per each PHQ-9 item were tabulated while the overall severity level was graphed. An ordinal logistic regression model was used to assess significant predictors of depression severity among elderly study participants based on an adjusted odds ratio with a 95% confidence interval.

## Results

A total of 191 eligible elderly participants completed the study questionnaire. Participants ranged from 60 to 88 years, with a mean age of 65.4 ± 5.5 years old. 106 (55.5%) were female, 143 (47.9%) were married, and 41 (21.5%) were divorced/widowed. One hundred seventy-six (92.1%) participants lived with their families, and only 15 (7.9%) lived alone. As for their educational level, 37 (19.4%) had a high school education, 79 (41.4%) were university graduates, and 52 (27.2%) had post-gradate degrees. 25 (13.1%) participants reported a monthly income of less than 5,000 SR, 46 (24.1%) had a monthly income ranging between 5,000-10,000 SR, while 120 (62.8%) had more than 10,000 SR. The vast majority (90.1%) of participants were either not working or retired, and 19 (9.9%) were working (Table 1).

**Table 1:** Socio-demographic data of elderly study participants in Makkah, Saudi Arabia

Socio-demographic data	No	%
Age in years		
60-65	132	69.1%
66-70	33	17.3%
> 70	26	13.6%
Gender		
Male	85	44.5%
Female	106	55.5%
Marital status		
Single	7	3.7%
Married	143	74.9%
Divorced/widowed	41	21.5%
Living with		
With family	176	92.1%
Alone	15	7.9%
Educational level		
Illiterate	8	4.2%
Below high school	15	7.9%
High school	37	19.4%
Bachelor’s degree	79	41.4%
Post-graduate degree	52	27.2%
Monthly income		
< 5000 SR	25	13.1%
5000-10000 SR	46	24.1%
> 10000 SR	120	62.8%
Work		
Not working/retired	172	90.1%
Working	19	9.9%

Table 2 shows the medical data of the elderly study participants residing in Makkah, Saudi Arabia. A total of 89 (46.6%) participants were hypertensive, 81 (42.4%) were diabetic, 33 (17.3%) complained of hypothyroidism, 15 (7.9%) had CVDs, and 12 (6.3%) had psychological disorders. A total of 45 (23.6%) participants had no chronic health problems. A total of 32 (16.8%) participants were diagnosed with depression and were prescribed medications for depression by doctors.

**Table 2:** Medical data among elderly study participants in Makkah, Saudi Arabia

Medical data	No	%
Chronic diseases		
Hypertensive	89	46.6%
Diabetic	81	42.4%
Others	34	17.8%
Hypothyroidism	33	17.3%
CVDs	15	7.9%
Psychological disorders	12	6.3%
Disability	11	5.8%
Tumor	8	4.2%
Stroke	7	3.7%
None	45	23.6%
Do you have cognitive impairment (memory problem)?		
No	148	77.5%
Yes	43	22.5%
Have you ever been diagnosed with depression previously?		
No	159	83.2%
Yes	32	16.8%
Has your doctor ever prescribed medications for depression?		
No	159	83.2%
Yes	32	16.8%

The PHQ-9 responses are illustrated in Table 3. 70.7% of participants felt tired or had little energy, 57.6% had trouble falling or staying asleep or sleeping too much, 53.4% experienced little interest or pleasure in doing things, 47.6% felt down, depressed, or hopeless, and 45% felt bad about themself, or that they were a failure or let themself or their family down. Only 19.4% thought that they would be better off dead or had thoughts of hurting themself in some way.

**Table 3:** PHQ-9 responses among elderly study participants in Makkah, Saudi Arabia

PHQ-9	Not at all		Several days		More than half days		Nearly every day	
	No	%	No	%	No	%	No	%
Little interest or pleasure in doing things?	89	46.6%	50	26.2%	27	14.1%	25	13.1%
Feeling down, depressed, or hopeless?	100	52.4%	50	26.2%	24	12.6%	17	8.9%
Trouble falling or staying asleep, or sleeping too much?	81	42.4%	45	23.6%	25	13.1%	40	20.9%
Feeling tired or having little energy?	56	29.3%	60	31.4%	31	16.2%	44	23.0%
Poor appetite or overeating?	94	49.2%	45	23.6%	20	10.5%	32	16.8%
Feeling bad about yourself – or that you are a failure or have let yourself or your family down?	105	55.0%	50	26.2%	14	7.3%	22	11.5%
Trouble concentrating on things, such as reading the newspaper or watching television?	115	60.2%	32	16.8%	19	9.9%	25	13.1%
Moving or speaking so slowly that other people could have noticed? Or so fidgety or restless that you have been moving a lot more than usual?	128	67.0%	29	15.2%	19	9.9%	15	7.9%
Thoughts that you would be better off dead, or thoughts of hurting yourself in some way?	154	80.6%	18	9.4%	8	4.2%	11	5.8%

**Figure 1:**
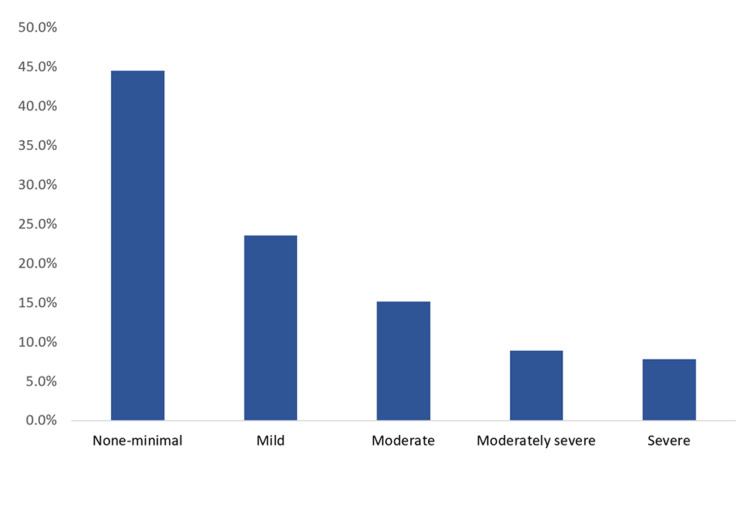
Prevalence of depression and its severity among elderly study participants residing in Makkah, Saudi Arabia

As illustrated in Figure 1, the prevalence of depression and its severity was the following: 85 (44.5%) participants had no or minimal depression, 45 (23.5%) had mild depression, 29 (15.2%) had moderate depression, and 32 (16.8%) had moderately severe and severe depression. The prevalence of major depression per the included sample was 61 (32%).

Results yielded from an ordinal logistics regression analysis for predictors of depression severity among elderly residents are shown in Table 4. Among all included factors, elderly participants with insomnia showed nearly a tripled risk of experiencing more severe depression (OR=2.74; 95% CI: 1.42-5.28). Likewise, elderly participants with a cognitive disorder were 2.5 times more likely to have more severe depression than those without (OR=2.63; 95% CI: 1.29-5.40). Elderly participants suffering from chronic health problems had 2.6 times more likelihood of higher depression compared to those without (OR=2.62; 95% CI: 1.11-6.14) while keeping all other factors constant.

**Table 4:** Ordinal logistics regression analysis for predictors of depression severity among elderly study participants residing in Makkah, Saudi Arabia AOR: Adjusted odds ratio; SE: Standard error; CI: Confidence interval * P < 0.05 (significant)

Factors	Coef.	AOR	SE	z	P>z	95% CI	
Age in years	0.03	1.03	0.03	1.10	0.273	0.97	1.09
Female gender	-0.47	0.62	0.33	-1.42	0.155	0.33	1.20
Divorced/widowed	0.35	1.42	0.40	0.88	0.381	0.65	3.11
Living alone	-0.26	0.77	0.60	-0.43	0.668	0.24	2.51
High education	-0.36	0.70	0.18	-1.98	0.047*	0.49	1.00
High income	0.09	1.09	0.27	0.34	0.736	0.65	1.84
Working	0.20	1.23	0.52	0.39	0.695	0.44	3.38
Experiencing pain	0.29	1.34	0.38	0.78	0.438	0.64	2.80
Insomnia	1.01	2.74	0.33	3.01	0.003*	1.42	5.28
Chronic disease	0.96	2.62	0.44	2.21	0.027*	1.11	6.14
Cognitive disorder	0.97	2.63	0.37	2.65	0.008*	1.29	5.40
History of depression	0.02	1.02	0.70	0.02	0.981	0.26	4.02
Medication for depression	0.16	1.18	0.68	0.24	0.812	0.31	4.47

## Discussion

As far as we know, our study is the first few studies to screen depression prevalence and its related risk factors among older Saudi residents in Makkah, Saudi Arabia. Based on PHQ-9, our findings indicated that 32% of elderly participants possibly had a depressive disorder. The prevalence of depression in our study was higher than that in a previous study conducted in Saudi Arabia, which was 12% [7]. The prevalence rates in previous studies may vary due to differences in diagnostic criteria and methods [15]. In addition, it is well known that the prevalence of late-life depression is underdiagnosed for several reasons, including the fact that depressive disorders manifest differently in geriatric populations, as well as the fact that depressive symptoms are often regarded as an inevitable part of aging [16]. According to our study, only 7.9% of participants were classified as having severe depression, while the most common subtype of depression was mild depression (23.5%). Our findings are slightly lower than those of another Saudi study that reported 28.2% of participants had mild depression [7]. A low prevalence of severe depression in our study can be attributed to religious, cultural, and strong social ties [7].

In our study, we used PHQ-9, a questionnaire designed to screen for depression in primary care and other community settings. Moreover, PHQ-9 is the most accessible and reliable screening tool [17]. Evidence has shown that this test is highly sensitive and specific [18], making it the gold-standard screening test for the diagnosis of depression in primary care patients [19-20]. Further research has also demonstrated its validity as a screening tool for the broader community [21]. Currently, the utilization of this tool has become prevalent to assess depression among individuals in the general community, as well as within primary care settings. 

We found that 46.6% of participants were hypertensive, 42.4% were diabetic, 17.3% complained of hypothyroidism, and 7.9% had CVDs. In line with other studies, our analysis revealed that participants with comorbid chronic diseases were significantly more likely to experience depression than those without chronic diseases [22-25]. On the other hand, the presence of chronic diseases did not contribute to the incidence of depression [26]. Among older adults, depression can be the first symptom of a variety of medical conditions, and these conditions often coexist. Also, depressive symptoms are associated with negative health outcomes such as heart disease [4]. Because chronic diseases can cause depression, major depressive disorder cannot be diagnosed when symptoms are caused directly by medical conditions [27]. There is a double risk of comorbid depression in diabetics [26], and a randomized study found that improving glycemic control corresponded to an improvement in depression scores [28]. A meta-analysis demonstrated statistically significant links between depression and diabetes complications [29]. The relationship between depression and blood pressure is a complex issue, and published reports describe higher blood pressure and higher incident hypertension in depressed patients [30]. Another systematic review and meta-analysis study found a moderate association between hypothyroidism and clinical depression [31]. Symptoms of hypothyroidism and depression partly overlap, and thyroid hormones may play some role in brain function, and immunologic processes may be involved between autoimmune thyroiditis and depression [31].

We found insomnia to be a risk factor for depression, which is in line with other studies [23]. Insomnia is not only related to developing dysthymia and depression in older adults but the persistence of insomnia has also been linked to the persistence of depression [32]. Moreover, sleep disturbances were an independent risk factor for relapse of depression in older adults in remission from depression [33].

Other studies have also found cognitive impairment to be a risk factor for depression [34-37]. A study found that over three years, irreversible dementia is more common in depressed patients with dementia syndrome of depression (43%). The risk of dementia is two-fold higher with late-onset depression, according to two systematic meta-analyses of high-quality studies. However, no research has determined whether depression is a risk factor for dementia or a prodromal condition [34].

A strength of our study is that it has provided insight into the frequency of depression, which was obtained from a community elderly population, and depression was assessed using the widely accepted screening tool PHQ-9.

Limitations of the study

This pilot study used a small size sample of the population, which limited our study, so future studies may use larger samples. Furthermore, the study's results may have been underestimated since participants who answered the questionnaire were likely to be in better health and more capable of navigating the web. Older people with illnesses or those unable to access the internet were less likely to complete the questionnaire. Furthermore, using a "self-report" questionnaire is considered as another limitation. The accuracy of the PHQ-9 test is theoretically lower in self-report compared to others, such as semi-interview and full interview PHQ-9. Also, the credibility of information in some aspects, such as underlying diseases, is also lower compared to medical records, especially in the elderly (partly due to the nature of this specific population, such as recall bias and cognitive decline).

## Conclusions

Depression is common among the elderly population of Makkah, Saudi Arabia. They are more likely to suffer from mild and moderate than from severe depression. To improve quality of life and avoid mental health deterioration, primary care professionals must be aware of detecting and treating depression in older patients. Our findings can be used to build successful community initiatives to help older persons manage prevalent mental health illnesses, including depression. Furthermore, depression screening and treatment by health care professionals for old adults in medical settings is highly recommended.
